# Effectiveness of an Educational Intervention With Home Visits and Telephone Follow‐Up on Knowledge and Quality of Life in Patients With Heart Failure: A Randomized Clinical Trial

**DOI:** 10.1111/nhs.70309

**Published:** 2026-02-16

**Authors:** Cleidinaldo Ribeiro de Goes Marques, Eduesley Santana Santos, Luciane Souza da Silva, Eryck Araujo de Castro Guimarães, Tarcisio Gois dos Santos, Vinícius Barbosa dos Santos Sales, Wanessa Alves Silva, Antônio Carlos Sobral Sousa

**Affiliations:** ^1^ Health Education Department Federal University of Sergipe Aracaju Brazil; ^2^ Federal University of Sergipe Aracaju Brazil; ^3^ Health Education Department Federal University of Sergipe Lagarto Brazil; ^4^ Nursing Department Tiradentes University Aracaju Brazil; ^5^ Nurse Anesthesia Student Mayo Clinic School of Health Sciences Rochester Minnesota USA; ^6^ Federal University of Sergipe Lagarto Brazil; ^7^ Nursing Department Federal University of Sergipe Lagarto Brazil

**Keywords:** health education, heart failure, knowledge, nursing care, quality of life

## Abstract

Cardiovascular diseases, particularly heart failure (HF), are leading causes of morbidity and mortality worldwide. Limited patient knowledge about HF is associated with poor treatment adherence and worse health outcomes, emphasizing the importance of educational interventions. This randomized clinical trial evaluated the effectiveness of a nurse‐led educational intervention combining home visits and telephone follow‐up on patients' knowledge and quality of life (QoL). Conducted in six Brazilian hospitals, the study included 120 patients hospitalized with decompensated HF and randomly assigned to a control group (CG) or an intervention group (IG). The IG received structured education at discharge, followed by home visits on days 7, 30, and 60 and telephone follow‐up on days 15 and 45, while the CG received usual care. Outcomes were assessed at 60 days. At this time point, adequate HF knowledge was observed in 82% of patients in the IG compared with 38% in the CG, and good QoL in 73% versus 33%, respectively (*p* < 0.001). The intervention was effective in improving knowledge and QoL in patients with HF.

## Key Points

- A nurse‐led educational intervention involving home visits and telephone follow‐up was associated with greater disease‐related knowledge and improved quality of life among patients with heart failure compared with usual care.
- Educational interventions extending beyond the hospitalization period contribute to sustained improvements in patient‐reported outcomes.

## 1 Introduction

Cardiovascular Diseases (CVDs) are among the leading causes of illness worldwide. Within this group, Heart Failure (HF) stands out as the final stage of most heart diseases and affects ~26 million people globally (Cestari et al. 2022). Due to its high morbidity and mortality, extensive healthcare resource use, prolonged hospital stays, and frequent readmissions, HF is considered a public health issue and a recurrent topic in research (Lawson et al. 2021).

Recent studies emphasize that patient knowledge of HF causes, symptoms, and treatment impacts their ability to recognize signs of worsening and adhere to medical recommendations, leading to improved clinical outcomes (Shoshima et al. 2023; Shi et al. 2024). Furthermore, HF's clinical manifestations often limit daily activities and require significant lifestyle changes, which substantially affect quality of life (QoL) (Heart Failure Guideline Coordinating Committee 2018).

Health education emerges as a vital tool to enhance patient engagement in self‐care strategies, improving disease management and reducing hospitalizations (Yu et al. 2022). In this scenario, healthcare professionals must adopt accessible and tailored approaches to promote a specific understanding of HF and its treatment, considering patients' age, comorbidities, socioeconomic status, and education level (Alnomasy and Still 2023).

Educational interventions combining Home Visits (HV) with telephone support have shown great potential in promoting HF patient self‐care. HVs enable healthcare professionals to tailor guidance to the patient's living conditions, identify barriers to treatment, and provide practical solutions (Dalai et al. 2021). Telephone follow‐ups complement HV by reinforcing information, offering emotional support, and addressing concerns promptly (Carreño et al. 2023).

Therefore, the present study aims to evaluate the effectiveness of an educational intervention based on home visits and telephone assessments on patients' disease knowledge and quality of life.

## 2 Method

### 2.1 Study Type

This is a Randomized Controlled Trial (RCT) guided by the CONSORT checklist and conducted in six hospitals in a Brazilian state. The project is registered in the Brazilian Registry of Clinical Trials (REBEC) under the number RBR‐8dymr8.

### 2.2 Population and Sample Size Planning

The population consists of patients admitted with a diagnosis of decompensated heart failure (HF). The sample size estimate was based on a two‐tailed analysis, with an alpha error of 0.05 and 80% power for an absolute incidence of 36% of the combined primary outcome of readmission and death, assuming the treatment would reduce the incidence to 13% (Crespo‐Leiro et al. 2018). It was estimated that 108 patients were required to achieve statistical power (54 in each group). Adding 10% to account for potential losses during the study, the total reached 120 patients, with 60 patients in each group. There was no equitable distribution of patients among the six hospitals.

### 2.3 Eligibility Criteria

Patients of both sexes aged 18 years or older, diagnosed with heart failure (HF) with systolic dysfunction and hospitalized due to clinical decompensation, were eligible for inclusion. Exclusion criteria were: absence of telephone contact; visual or hearing impairments and/or mobility limitations that interfered with activities of daily living; New York Heart Association (NYHA) functional class IV; participation in another study addressing the same topic; hospitalization for surgical procedures unrelated to HF; residence located more than 50 km from any of the participating hospitals; and cognitive impairment assessed using the Mini‐Mental State Examination (MMSE). The following MMSE cutoff scores were applied: ≥ 13 points for illiterate patients, ≥ 18 points for those with 1–7 years of schooling, and ≥ 26 points for individuals with eight or more years of formal education (Lenardt 2009).

### 2.4 Data Collection and Variable Analysis Tools

#### 2.4.1 Sociodemographic and Clinical Questionnaire

For the collection of sociodemographic and clinical data, an adapted instrument was used (Mussi et al. 2013), divided into two sections: (1) sociodemographic data and (2) clinical data.

#### 2.4.2 Health‐Related Quality‐of‐Life Questionnaire

The Brazilian version of the Minnesota Living With Heart Failure Questionnaire was used to measure quality of life (QoL). For this study, QoL was categorized as good (≤ 26 points), moderate (27–45 points), and poor (> 45 points), according to established cutoffs described in the literature and commonly applied in clinical and research settings to facilitate interpretation of health‐related quality‐of‐life outcomes in patients with heart failure (Carvalho et al. 2020).

#### 2.4.3 HF Knowledge Questionnaire

To assess knowledge about heart failure (HF), a validated questionnaire translated into Portuguese was applied, consisting of 14 objective questions. Knowledge was considered adequate when the patient achieved at least 70% correct answers, a cutoff commonly used in health education research to indicate a satisfactory level of content mastery and sufficient understanding to support appropriate self‐care behaviors. This threshold has been widely applied in studies evaluating disease‐related knowledge in patients with chronic conditions, including HF, allowing comparability across studies and clinical interpretability of results (Rabelo et al. 2011).

#### 2.4.4 Educational Booklet on Heart Failure

The booklet titled “Guidelines for Patients with Heart Failure Post‐Hospital Discharge” was used during face‐to‐face assessments for the intervention group (IG) and the control group (CG) (only after the completion of their assessments) (Marques et al. 2023). The topics covered in the booklet included: heart function, basic concepts of HF pathophysiology, causes, signs and symptoms of the disease, recommendations on physical activity, clinical warning signs, dietary habits, weight control, vaccinations, and pharmacological treatment.

#### 2.4.5 Self‐Care Management Instrument

Patients in the IG were given a tool created by the authors to monitor and record their weight (2 to 3 times per week), readmission occurrences, and medications in use. For patients in the CG, a form was provided specifically for recording readmissions.

#### 2.4.6 Telephone Consultation Instrument

Telephone consultations were conducted at pre‐established intervals using a standardized tool. Their purpose was to maintain active contact with patients, assess their current health status, and reinforce and supplement the recommendations provided in the educational booklet.

### 2.5 Data Collection System

Patients were recruited through daily visits to the inpatient wards of each institution. When eligible patients were identified and expressed willingness to participate in the study, the Informed Consent Form (ICF) was applied. Following this, the sociodemographic and clinical questionnaire and the MMSE were applied. Participants meeting the inclusion criteria were randomly assigned to the control group (CG) or intervention group (IG) using a simple sequence generated by computer on the website www.randomization.com. A researcher not involved in data analysis managed the randomization list. Subsequently, a nurse was called in to conduct the evaluation. The outcome assessor was unaware of group allocation.

#### 2.5.1 Intervention Group (IG)

Participants allocated to the intervention group were followed by nurses through face‐to‐face consultations and telephone consultations. Face‐to‐face nursing consultations were conducted at four time points: in the hospital during the hospitalization period and at 7, 30, and 60 days after discharge, at the participants' homes. Each face‐to‐face consultation lasted ~90 min. Telephone consultations were conducted at 15 and 45 days after discharge, with an average duration of 15 min.

During the in‐person nursing consultations, a cardiopulmonary physical examination was performed, the HF Knowledge Questionnaire and the Quality‐of‐Life Questionnaire were applied, and the educational intervention was carried out using the validated booklet for this study. All instruments were applied before the educational intervention to avoid influencing the assessment of the patient's prior knowledge.

The educational booklet was read and explained in detail, linking its contents to the patient's reality. In cases of questions or difficulties in understanding, the content was repeated, and the explanations were reinforced. Regarding the self‐care management tool, the nurse filled out the section for recording the patient's daily medications, providing guidance on their purpose and use and instructed the patient on the need to record their weight (2 to 3 times per week) and any readmissions in the same document.

As for the telephone consultations, they were conducted on the 15th and 45th days post‐discharge with an educational purpose. For this, the nurse used a specific tool and followed a call protocol, discussing the content printed in the educational booklets in a dialogic format to reinforce the guidance provided during the in‐person consultations. The proposed average time to reinforce the educational instructions and evaluate behavioral changes was 15 min.

#### 2.5.2 Control Group (CG)

Patients in the control group (CG) were evaluated in person before hospital discharge. During this consultation, a cardiopulmonary assessment was conducted, and the HF Knowledge and Quality‐of‐Life Questionnaires were applied. Patients in the control group (CG) did not receive educational intervention from the research nurse. At the end of the nursing consultation, they were informed about follow‐up contact at 30 days post‐discharge and were given a tool to record the number of readmissions. At 30 days post‐discharge, a telephone contact was made to maintain the participant's active engagement in the study and to assess their clinical condition. At 60 days post‐discharge, a new in‐person consultation was conducted, following the same procedures as the initial consultation. After the final evaluation and completion of the instruments, CG patients received guidance on heart failure using the educational booklet.

### 2.6 Ethical Aspects

The study was approved by the Research Ethics Committee (Approval Number: 2897628). All participants provided their consent by signing the Informed Consent Form (ICF). The evaluations were conducted following the recommendations of the Declaration of Helsinki and in compliance with Brazil's General Data Protection Law. There was no use of Artificial Intelligence Generated Content (AIGC) tools and others based on large language models (LLMs).

### 2.7 Data Analysis

Descriptive measures were used to summarize the characteristics of the variables and provide an overview of the collected data. The Chi‐square test and Fisher's exact test were applied to assess associations between categorical variables, according to expected cell frequencies. Data normality was evaluated using the Shapiro–Wilk test. For comparisons between two independent groups with non‐normally distributed continuous variables, the Wilcoxon–Mann–Whitney test was used.

Given the longitudinal design with repeated measurements, statistical methods were selected according to the distribution and scale of the outcome variables. For categorical and ordinal outcomes, including the classification of knowledge and quality of life (QoL), Generalized Estimating Equations (GEE) with binomial or ordinal distributions and a logit link function were applied to account for within‐subject correlation, provide population‐averaged estimates, and allow the inclusion of participants with incomplete follow‐up. For continuous outcomes with approximately normal distribution, such as total knowledge and QoL scores, Repeated Measures Analysis of Variance (ANOVA) was used to evaluate changes over time and differences between groups.

Although the original sample size calculation was based on a composite clinical outcome (readmission and death), post hoc power analyses were conducted for the primary outcomes of this study (knowledge and QoL) using the final analyzed sample sizes, a two‐sided significance level of 5%, and the observed effect sizes at 60 days. The achieved statistical power exceeded 99% for knowledge and 95% for QoL, indicating that the final sample size was adequate to detect clinically meaningful differences between groups.

All statistical analyses were performed using the R programming environment (version 4.3.2) (R Core Team 2023), and statistical significance was set at *p* < 0.05.

## 3 Results

The data presented in this study correspond to the period from September 2022 to February 2025. During this time, 430 patients were hospitalized for decompensated heart failure (HF) in the six participating hospitals. Of these 430 patients, 276 consented to participate in the study, 75 refused, and 79 were excluded for various reasons (death, clinical deterioration preventing data collection, discharge before evaluation, among others). After the application of the clinical and sociodemographic questionnaire and the Mini‐Mental State Examination (MMSE), 156 patients were excluded for not meeting the inclusion criteria. A total of 120 patients were randomly assigned, with 60 in the intervention group (IG) and 60 in the control group (CG).

**FIGURE 1:**
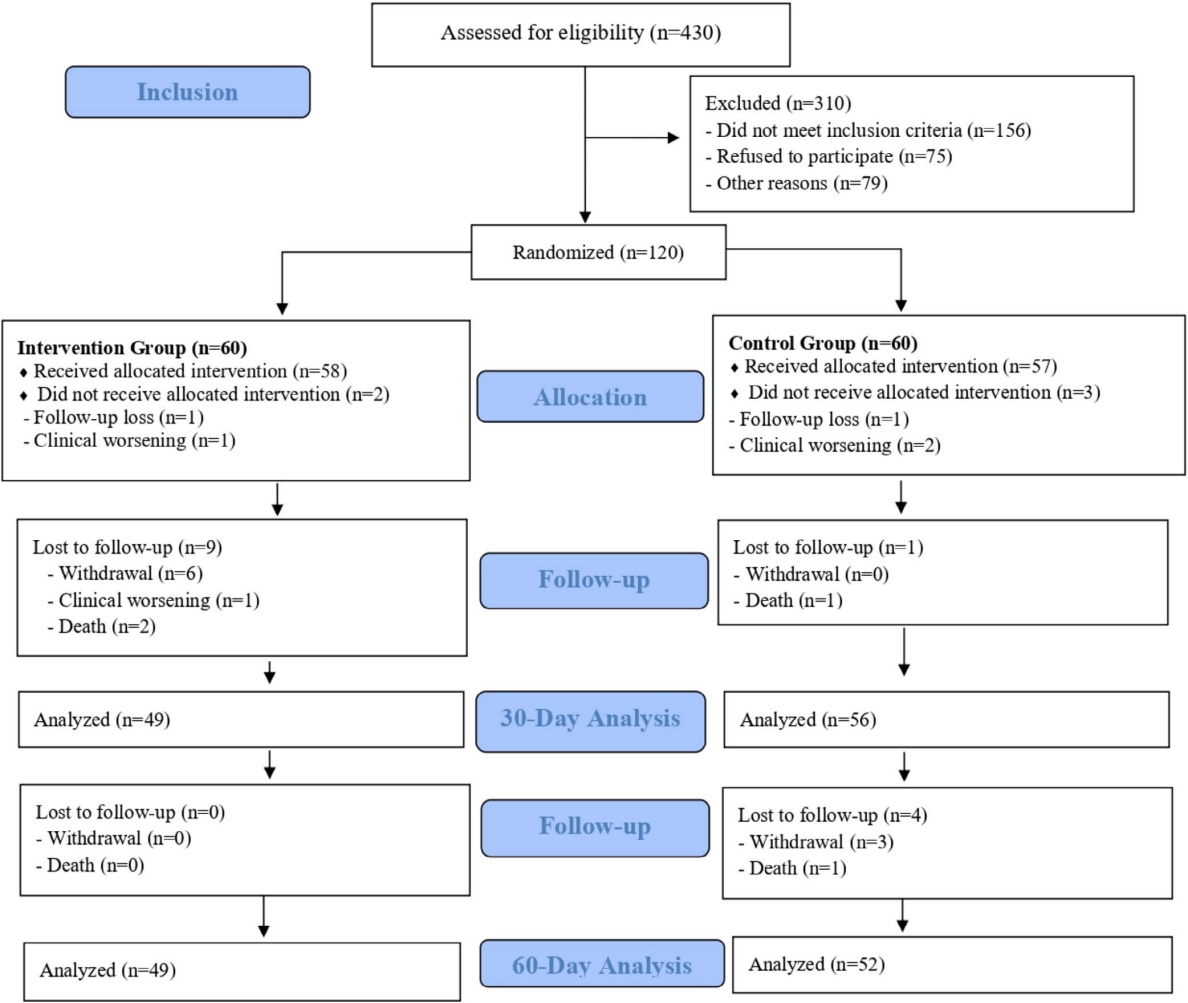
CONSORT flowchart.

From the 120 randomized patients, 101 completed the 60‐day follow‐up after hospital discharge, with 52 in the control group (CG) and 49 in the intervention group (IG). During the study period, there were eight losses in the control group (CG) (two deaths due to cardiac complications related to heart failure decompensation, four withdrawals, and two cases of clinical deterioration resulting from low cardiac output and cardiopulmonary decompensation) and 11 losses in the intervention group (IG) (two deaths due to cardiac complications related to heart failure decompensation, seven withdrawals, and two cases of clinical deterioration resulting from low cardiac output and cardiopulmonary decompensation). Figure 1 presents the CONSORT flowchart.

The sociodemographic characteristics are presented in Table 1. In both groups there was a predominance of men (57%), with a mean age of 66 years, mixed race (CG 60% vs. 45% IG), and married (52%).

**TABLE 1:** Clinical and sociodemographic characteristics.

Characteristics	Group		*p* value
	Control, *N* = 60	Intervention, *N* = 60	
Age			0.765^c^
Mean (Standard Deviation)	66 (15)	66 (16)	
Sex (Male), *n*/*N* (%)	34/60 (57%)	34/60 (57%)	> 0.999^b^
Race, *n*/*N* (%)			0.238^b^
White	14/60 (23%)	21/60 (35%)	
Black	10/60 (17%)	12/60 (20%)	
Mixed	36/60 (60%)	27/60 (45%)	
Education level, *n*/*N* (%)			0.023^b^
Illiterate	12/60 (20%)	4/60 (6%)	
1–7 years	16/60 (27%)	28/60 (47%)	
8 or more years	32/60 (53%)	28/60 (47%)	
Marital status, *n*/*N* (%)			0.605^b^
Married	31/60 (52%)	31/60 (52%)	
Single	9/60 (15%)	14/60 (23%)	
Widowed	14/60 (23%)	11/60 (18%)	
Divorced	6/60 (10%)	4/60 (7%)	
Duration of HF, *n*/*N* (%)			0.707^b^
Up to 1 year	16/60 (27%)	20/60 (33%)	
1–5 years	19/60 (32%)	14/60 (23%)	
> 5 years	19/60 (32%)	21/60 (35%)	
Unknown	6/60 (10%)	5/60 (9%)	
Etiology, *n*/*N* (%)			0.341^a^
Ischemic	19/60 (32%)	27/60 (45%)	
Hypertensive	3/60 (5%)	7/60 (12%)	
Valvular	19/60 (32%)	15/60 (25%)	
Alcoholic	1/60 (1%)	1/60 (1%)	
Idiopathic	6/60 (10%)	2/60 (4%)	
Chagasic	3/60 (5%)	1/60 (1%)	
Unidentified	9/60 (15%)	7/60 (12%)	
Functional class (NYHA), *n*/*N* (%)			0.734^b^
I	15/60 (25%)	13/60 (22%)	
II	27/60 (45%)	25/60 (42%)	
III	18/60 (30%)	22/60 (37%)	
Previous HF Hospitalizations, *n*/*N* (%)	30/60 (50%)	33/60 (55%)	0.583^b^
Number of Previous HF Hospitalizations			0.125^c^
Mean (Standard Deviation)	2.7 (1.9)	2.2 (1.9)	
Comorbidities			
HTN, *n*/*N* (%)	45/60 (75%)	52/60 (87%)	0.104^b^
DM, *n*/*N* (%)	28/60 (47%)	26/60 (43%)	0.714^b^
Dyslipidemia, *n*/*N* (%)	20/60 (33%)	19/60 (32%)	0.845^b^
Heart Disease, *n*/*N* (%)	25/60 (42%)	20/60 (33%)	0.346^b^
Angioplasty, *n*/*N* (%)	23/60 (38%)	27/60 (45%)	0.459^b^
Number of Angioplasties			0.603^c^
Mean (Standard Deviation)	1.4 (0.8)	1.8 (1.5)	
Alcohol Consumption, *n*/*N* (%)	14/60 (23%)	13/60 (22%)	0.629^b^
Former Drinker, *n*/*N* (%)	26/60 (43%)	22/60 (37%)	
Physical Activity, *n*/*N* (%)	16/60 (27%)	16/60 (27%)	0.956^b^
Vaccination Schedule, *n*/*N* (%)	49/60 (82%)	46/60 (77%)	0.500^b^
MEEM			0.987^b^
Mean (Standard Deviation)	25.3 (4.1)	25.0 (4.5)	
MEEM classification, *n*/*N* (%)			0.757^c^
Normal	36/60 (60%)	37/60 (62%)	
Mild	14/60 (23%)	11/60 (18%)	
Moderate	10/60 (17%)	12/60 (20%)	
MEDICATIONS IN USE			
Antiplatelet Agent, *n*/*N* (%)	15/60 (25%)	21/60 (35%)	0.232^b^
Beta Blocker, *n*/*N* (%)	37/60 (62%)	35/60 (58%)	0.709^b^
Thiazide diuretic, *n*/*n* (%)	6/60 (10%)	9/60 (15%)	0.408^b^
Statin, *n*/*n* (%)	40/60 (67%)	28/60 (47%)	**0.027** ^b^
Anticoagulant, *n*/*n* (%)	9/60 (15%)	4/60 (7%)	0.142^b^
Antihypertensive, *n*/*n* (%)	19/60 (32%)	27/60 (45%)	0.133^b^
Loop diuretic, *n*/*n* (%)	45/60 (75%)	46/60 (77%)	0.831^b^
Potassium sparing diuretic, *n*/*n* (%)	32/60 (53%)	18/60 (30%)	**0.010** ^b^
Vasodilator, *n*/*n* (%)	11/60 (18%)	10/60 (17%)	0.810^b^
LVEF %			0.116^c^
Mean (Standard Deviation)	42 (16)	47 (18)	
LVEF Classification, *n*/*N* (%)			0.141^b^
Preserved	18/57 (32%)	27/56 (48%)	
Intermediate	13/57 (23%)	7/56 (13%)	
Reduced	26/57 (46%)	22/56 (39%)	

Abbreviations: %, Percentage; DM, Diabetes Mellitus; HF, Heart Failure; HTN, Systemic Arterial Hypertension; IQR, Interquartile Range; MEEM, Mini‐Mental State Examination; MMSE, Mini‐Mental State Examination; *n*, Absolute Frequency; *N*, Valid Data; SD, Standard Deviation.

^a^ Fisher's exact test.

^b^ Pearson's Chi‐squared test.

^c^ Wilcoxon rank sum test.

Regarding clinical characteristics, in the control group (CG), the following predominated: time since diagnosis of the disease greater than 1 year (64%), valvular or ischemic etiology (32%), NYHA functional class II (45%), a mean left ventricular ejection fraction (LVEF) of 42 ± 16% and predominance of reduced classification (46%). In contrast, the intervention group (IG) showed a predominance of a diagnosis duration of at least 5 years (35%), ischemic etiology (45%), NYHA functional class II (42%), a mean LVEF of 47 ± 18% with a predominance of preserved classification (48%), and hospitalization within the past year for HF in 55% of the patients.

Regarding current and past health history, lifestyle habits, and comorbidities, there was a predominance of hypertension (HTN) (75% in CG vs. 87% in IG, *p* = 0.104) and diabetes mellitus (DM) (47% in CG vs. 43% in IG, *p* = 0.714). The majority had not undergone angioplasty (62% in CG vs. 55% in IG, *p* = 0.459), did not drink alcohol (23% in CG vs. 22% in IG, *p* = 0.629), were former drinkers (43% in CG vs. 37% in IG), and did not engage in physical activity (73% in CG and IG, *p* = 0.956).

The pharmacological information and laboratory tests are detailed in Table 1. Similarities were observed in the most commonly used medications among patients in both groups, with a predominance of beta‐blockers, statins, and diuretics.

During hospitalization, most patients in both groups demonstrated inadequate prior knowledge of heart failure (HF) (60% in CG vs. 57% in IG; *p* = 0.765). By the end of the follow‐up, a higher proportion of CG patients still had inadequate knowledge (62%, *n* = 32). In contrast, IG patients showed a greater proportion of adequate knowledge (82%, *n* = 40), highlighting the significant impact of the educational intervention compared with the CG (*p* < 0.001) (Table 2).

**TABLE 2:** Changes in knowledge of heart failure and comparison between the two groups.

Characteristics	Group		*p* value
	Control, *N* = 60	Intervention, *N* = 60	
Knowledge_in hospital__Total			0.975^a^
Mean (Standard Deviation)	61 (16)	62 (17)	
Knowledge_in hospital_Classification, *n*/*N* (%)			0.765^c^
Inadequate	34/57 (60%)	33/58 (57%)	
Adequate	23/57 (40%)	25/58 (43%)	
Knowledge_at 7 days_Total			
Mean (Standard Deviation)	NA (NA)	71 (19)	
Knowledge_at 7 days_Classification, *n*/*N* (%)			
Inadequate	NA (NA)	22/51 (43%)	
Adequate	NA (NA)	29/51 (57%)	
Knowledge_at 30 days_Total			
Mean (Standard Deviation)	NA (NA)	77 (18)	
Knowledge_at 30 days_Classification, *n*/*N* (%)			
Inadequate	NA (NA)	15/49 (31%)	
Adequate	NA (NA)	34/49 (69%)	
Knowledge_at 60 days_Total			< 0.001^a^
Mean (Standard Deviation)	63 (14)	81 (18)	
Knowledge_at 60 days_Classification, *n*/*N* (%)			< 0.001^b^
Inadequate	32/52 (62%)	9/49 (18%)	
Adequate	20/52 (38%)	40/49 (82%)	

Abbreviations: %, Percentage; Av, Evaluation; IQR, Interquartile Range; *n*, Absolute Frequency; *N*, Valid Data; SD, Standard Deviation.

^a^ Wilcoxon Rank‐Sum Test.

^b^ Chi‐Square Test of Independence.

^c^ Fisher's Exact Test.

**FIGURE 2:**
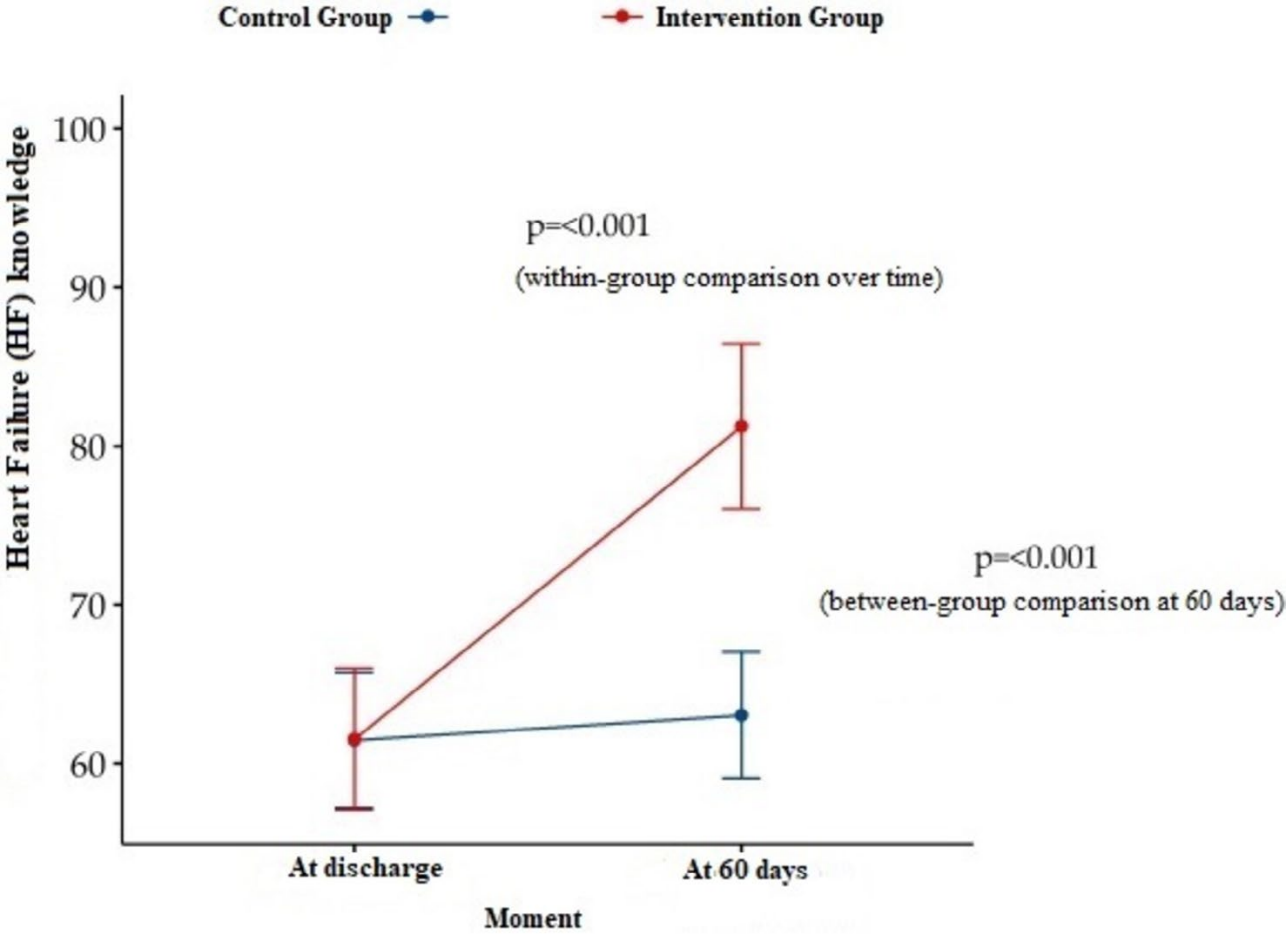
Changes in total scores for knowledge of heart failure.

Simultaneously, an increase in adequate knowledge was observed over the consultations conducted in the intervention group (IG), with a significant difference between the first and last consultations (*p* < 0.001) (Figure 2).

QoL was classified as poor, moderate, or good based on the scores achieved by patients during the questionnaire application. In both groups, poor QoL predominated during the hospitalization assessment (61% in the control group [CG] vs. 69% in the intervention group [IG]; *p* = 0.482). After the final evaluation, a higher proportion of good QoL was observed in the IG compared with the CG (73% vs. 33%, respectively), demonstrating a significant impact of the educational intervention on the QoL of IG patients (*p* < 0.001) (Table 3).

**TABLE 3‐:** Changes in Quality‐of‐Life and comparison between the two groups.

Characteristics	Group		*p* value
	Control, *N* = 60	Intervention, *N* = 60	
QOL_in hospital_Total			0.845^a^
Mean (Standard Deviation)	53 (21)	53 (18)	
QOL_in hospital_Classification, *n*/*N* (%)			0.482^b^
Poor	35/57 (61%)	40/58 (69%)	
Moderate	16/57 (28%)	15/58 (26%)	
Good	6/57 (11%)	3/58 (5%)	
QOL_at 7 days_Total			
Mean (Standard Deviation)	NA (NA)	33 (22)	
QOL_at 7 days_Classification, *n*/*n* (%)			
Poor	NA (NA)	16/51 (31%)	
Moderate	NA (NA)	16/51 (31%)	
Good	NA (NA)	19/51 (38%)	
QOL_at 30 days_Total			
Mean (Standard Deviation)	NA (NA)	19 (15)	
QOL_at 30 days_Classification, *n*/*N* (%)			
Poor	NA (NA)	2/49 (4%)	
Moderate	NA (NA)	13/49 (27%)	
Good	NA (NA)	34/49 (69%)	
QOL_at 60 days_Total			0.003^a^
Mean (Standard Deviation)	35 (27)	19 (17)	
QOL_at 60 days_Classification, *n*/*N* (%)			< 0.001^c^
Poor	21/52 (40%)	4/49 (8%)	
Moderate	14/52 (27%)	9/49 (19%)	
Good	17/52 (33%)	36/49 (73%)	

Abbreviations: %, Percentage; IQR, Interquartile Range; *n*, Absolute Frequency; *N*, Valid Data; QOL, Quality‐of‐Life; SD, Standard Deviation.

^a^ Wilcoxon Rank‐Sum Test.

^b^ Fisher's Exact Test.

^c^ Chi‐Square Test of Independence.

**FIGURE 3:**
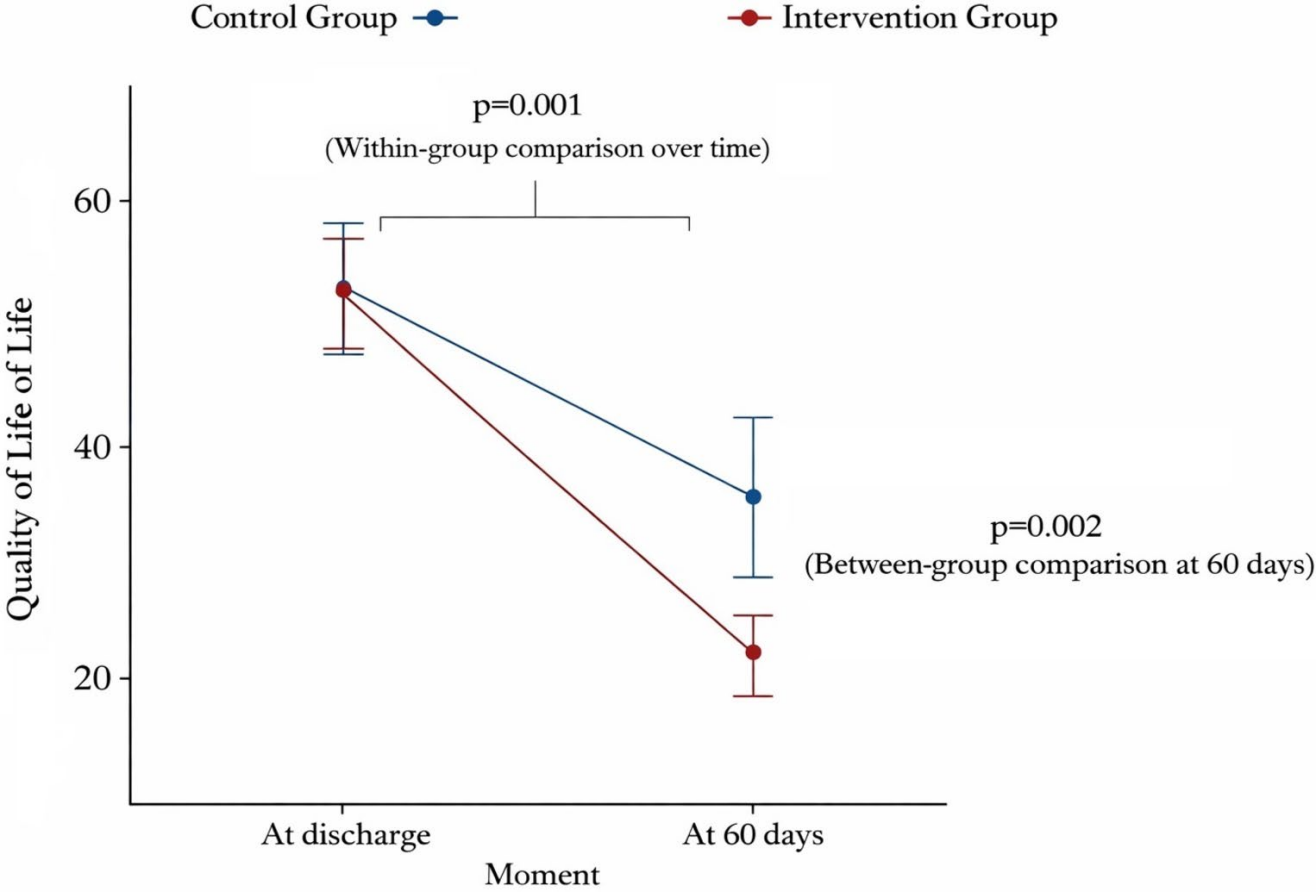
Changes in total scores for Quality‐of‐Life.

An improvement in the QoL of patients in the IG was observed in subsequent evaluations, with the classification of “Good QoL” increasing from 5% in the first consultation to 38%, 69%, and 73% in the following consultations, respectively. By the end of the study, a significant improvement in QoL was identified between the first and last consultations (*p* < 0.001) (Figure 3).

## 4 Discussion

In this randomized controlled trial (RCT), a nurse‐led educational strategy combining in‐person HVs and structured telephone consultations was implemented to improve patients' knowledge about HF and their QoL. Educational materials were specifically developed for this purpose and delivered exclusively by nurses. Four face‐to‐face nursing consultations were conducted for patients in the IG: one during hospitalization and three after discharge, in addition to two scheduled telephone consultations.

The in‐hospital consultation aimed to prepare patients for the discharge process, facilitating a safer transition from hospital to home care. Chang and Tung (2024) highlight the relevance of structured discharge planning, demonstrating that patients who received high‐quality discharge plans had significantly lower 30‐day readmission rates (24%) and mortality rates (5.1%) compared with those who received low‐quality discharge planning, who presented higher readmission (~31%) and mortality rates (around 7%). These findings reinforce that the quality of discharge planning is a determinant of early post‐discharge outcomes, supporting the emphasis placed on discharge preparation in the present intervention.

Post‐discharge, the remaining face‐to‐face consultations were conducted at the patients' homes. These home visits allowed nurses to tailor educational guidance to the patients' living conditions, focusing on adherence to pharmacological treatment, implementation of non‐pharmacological recommendations, and early identification of warning signs of clinical deterioration. Although multidisciplinary follow‐up is considered the gold standard in HF management, access to such care remains limited in many Brazilian healthcare settings, particularly at the primary healthcare level (Heart Failure Guideline Coordinating Committee 2018). In this context, nurse‐led home visits represent a feasible and effective alternative.

Telephone consultations were conducted at 15 and 45 days after discharge, following a standardized protocol regarding duration and content. These contacts aimed to reinforce the guidance provided during in‐person consultations, clarify doubts, and monitor adherence and symptom evolution. Previous studies have demonstrated that telephone follow‐up can improve treatment adherence, self‐care behaviors, and reduce hospital readmissions (Carreño et al. 2023; Moon et al. 2018).

The COVID‐19 pandemic further highlighted the importance of remote follow‐up strategies, particularly telephone‐based interventions, to ensure continuity of care. However, caution is required when relying exclusively on telephone interventions alone, as evidence suggests that their benefits are reduced when not combined with face‐to‐face contact. Limitations related to communication barriers, infrastructure, and professional training may further restrict the effectiveness of isolated telephone follow‐up (Camilo et al. 2021).

In this regard, the literature indicates that combined interventions, such as the integration of nurse‐led HV with structured telephone follow‐up, produce better outcomes than single interventions, including home visits alone or telephone contact alone. Combined strategies allow for comprehensive in‐person assessment and individualized education during HV, while telephone contacts reinforce guidance, promote continuity, and enable early problem identification. This synergistic approach may explain the superior outcomes observed in the present RCT (Heart Failure Guideline Coordinating Committee 2018).

Regarding the primary outcome of knowledge, a statistically significant improvement in knowledge scores was observed in the IG compared with the CG (*p* < 0.001), as well as a statistically significant increase between the first and final consultations within the IG (*p* < 0.001). Knowledge about HF is essential for effective disease management, as it enables patients to understand the etiology of the disease, recognize signs and symptoms, adhere to pharmacological and non‐pharmacological treatments, and identify situations that may precipitate clinical decompensation. In this context, disease‐related knowledge may influence clinical outcomes (e.g., symptom control and hospital readmissions), behavioral outcomes (e.g., adherence and self‐care), and quality‐of‐life outcomes, and therefore requires continuous assessment and reinforcement (Albino 2019).

Several factors may influence patients' level of knowledge. Shoshima et al. (2023) reported lower HF knowledge among older patients and those with lower income. Educational level is also a relevant determinant. Luz et al. (2020) demonstrated that patients with ≤ 8 years of schooling had significantly lower knowledge scores than those with > 8 years of education, and that cognitive decline was associated with poorer knowledge outcomes. In the present study, illiterate individuals and patients with ≤ 8 years of education were more frequent in the CG, which may have partially influenced baseline differences. However, this imbalance was addressed through individualized educational strategies in the IG.

To minimize the impact of educational disparities, the intervention was adapted to patients' educational background and cognitive abilities. For participants with low literacy or illiteracy, nurses used simplified language, visual resources, and images from the educational booklet to facilitate understanding, involving caregivers when necessary. These adaptations were applied consistently during home visits and telephone consultations, ensuring equitable access to information regardless of educational level.

Regarding QoL, a statistically significant improvement in QoL scores was observed in the IG compared with the CG (*p* < 0.001), as well as a statistically significant increase between the first and final consultations within the IG (*p* < 0.001). During hospitalization, QoL was classified as poor in both groups, reflecting the substantial physical, emotional, social, and spiritual burden imposed by HF (Carvalho et al. 2020). At the end of follow‐up, patients in the IG demonstrated meaningful improvements, likely associated with better symptom management, enhanced self‐care capacity, and improved clinical stability.

The effectiveness of this educational intervention appears to be related both to the combination of different intervention methods (in‐person home visits and telephone follow‐up) and to the frequency of contacts, which occurred approximately every 2 weeks over a two‐month period. Compared with previous studies that relied on a single modality or less frequent follow‐up, this approach provided continuous reinforcement, timely support, and sustained patient engagement. The integration of methods allowed nurses to address different needs across contexts, while the relatively short intervals between contacts may have enhanced learning retention and behavioral change, thereby contributing to the positive outcomes observed in this study (Alnomasy and Still 2023; Carreño et al. 2023; Silva et al. 2021).

## 5 Limitations

Some limitations must be acknowledged, including sample size, follow‐up duration, and the lack of involvement of other healthcare professionals. Despite these limitations, the benefits of the home visit (HV) strategy combined with telephone follow‐up were evident in the primary outcomes, particularly improvements in patient knowledge and quality of life. However, the wide territorial coverage, barriers to patient access, and limited financial resources may restrict the wider implementation of this intervention in routine clinical practice. Furthermore, this study did not compare the effects of a single intervention modality, such as telephone follow‐up alone, with those of a combined strategy involving home visits and telephone contacts; therefore, it cannot be concluded that the combined approach is superior to either modality when applied in isolation.

## 6 Conclusion

The findings of this study indicate that a nurse‐led educational intervention combining HV and structured telephone follow‐up significantly improved patients' knowledge about HF and health‐related quality of life, which were the primary outcomes evaluated. Although this study was not designed to directly assess long‐term clinical outcomes such as hospital readmissions or mortality, improved knowledge and quality of life are recognized intermediate outcomes that may support better self‐management in patients with chronic conditions such as HF.

In this context, the benefits identified in this study are primarily related to patient‐centered outcomes, including enhanced understanding of the disease, increased engagement in self‐care behaviors, and improved perceived quality of life. From a healthcare services perspective, the structured and protocol‐based nature of the intervention demonstrates its feasibility within nursing practice and its potential to support continuity of care after hospital discharge.

## 7 Relevance for Clinical Practice

The intervention model evaluated in this study provides practical elements that can be incorporated into outpatient and post‐discharge care for patients with heart failure. Specifically, the use of standardized educational materials, combined with scheduled home visits and telephone follow‐up conducted by nurses, offers a structured approach to reinforce patient education beyond the hospital setting. At the outpatient level, this model may be applied through:

- Scheduled nurse‐led follow‐up visits shortly after discharge to reassess symptoms, reinforce self‐care guidance, and address patient‐specific barriers.
- Structured telephone contacts to maintain continuity of education, monitor adherence‐related behaviors, and identify early warning signs requiring referral.
- Integration of self‐care monitoring tools (e.g., weight and medication records) into routine outpatient follow‐up to support patient engagement and clinical decision‐making.

By emphasizing structured education and continuity of nursing follow‐up, this approach may contribute to safer transitions from hospital to outpatient care and improved alignment between inpatient discharge planning and ongoing community‐based management.

## Author Contributions

C.R.G.M. contributed to conceptualization, investigation, methodology, validation, visualization, writing – original draft, writing – review and editing, and supervision. E.S.S. contributed to conceptualization, formal analysis, validation, visualization, writing – review and editing, and supervision. L.S.S. contributed to conceptualization, investigation, methodology, formal analysis, validation, visualization, writing – original draft, writing – review and editing, and supervision. E.A.C.G. contributed to conceptualization, investigation, methodology, formal analysis, validation, visualization, writing – original draft, and writing – review and editing. T.G.S. contributed to conceptualization, investigation, methodology, formal analysis, validation, visualization, writing – original draft, and writing – review and editing. V.B.S.S. contributed to conceptualization, investigation, methodology, formal analysis, validation, visualization, writing – original draft, and writing – review and editing. W.A.S. contributed to conceptualization, investigation, methodology, formal analysis, validation, visualization, writing – original draft, and writing – review and editing. A.C.S.S. contributed to conceptualization, methodology, formal analysis, validation, visualization, writing – original draft, writing – review and editing, and supervision. All authors approved the final version of the manuscript and agree to be accountable for all aspects of the work.

## Funding

The authors have nothing to report.

## Ethics Statement

The study was approved by the Research Ethics Committee (Approval Number: 2897628).

## Consent

All participants provided their consent by signing the Informed Consent Form (ICF). The evaluations were conducted following the recommendations of the Declaration of Helsinki and in compliance with Brazil's General Data Protection Law.

## Conflicts of Interest

The authors declare no conflicts of interest.

## Data Availability

The data that support the findings of this study are available from the corresponding author upon reasonable request.
